# Improving the Bioactivity and Antibiofilm Properties of Metallic Implant Materials via Controlled Surface Microdeformation

**DOI:** 10.1021/acsomega.4c07185

**Published:** 2024-10-10

**Authors:** Furkan Biçer, Sıdıka Mine Toker, Merve Nur Soykan, Burcu Türk Yılmaz, Bükay Yenice Gürsu, Onur Uysal

**Affiliations:** † Biotechnology and Biosafety Department, Eskisehir Osmangazi University, Eskisehir, 26040, Türkiye; ‡ Metallurgical and Materials Engineering Department, Eskisehir Osmangazi University, Eskisehir, 26040, Türkiye; § Cellular Therapy and Stem Cell Production Application, Research Centre (ESTEM) Eskisehir Osmangazi University, Eskisehir, 26040, Türkiye; ∥ Department of Stem Cell, Institute of Health Sciences, Eskisehir Osmangazi University, Eskisehir, 26040, Türkiye; ⊥ Central Research Laboratory Application and Research Center, Eskisehir Osmangazi University, Eskisehir, 26040, Türkiye

## Abstract

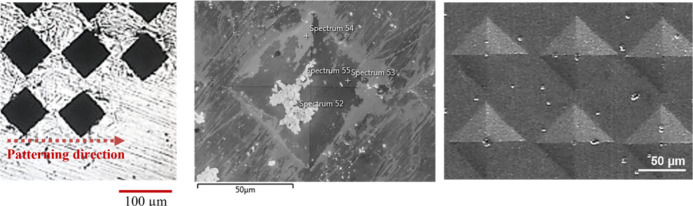

Although metallic implants provide most of the required properties for bone-related applications, especially orthopedic implants, insufficient osseointegration, which may lead to loosening of the implant or prolonged healing time, is still an issue to be resolved. Osseointegration can be improved via application of various surface treatments on the metal surface. The current study focuses on a novel surface microdeformation method, which enables the formation of controlled surface patterns of various parameters. With this purpose, a surface microdeformation procedure was applied on 316L stainless steel surfaces, forming four different patterns which affected various surface parameters such as roughness, surface energy, dislocation activities close to the surface, and wettability. Static immersion tests in a simulated body fluid (SBF) environment showed that modifying the surface parameters via controlled surface patterning promoted the formation of a stable oxide layer and calcium-phosphate (CaP) deposition on the metal surfaces, improving bioactivity. Moreover, the higher amount of CaP deposition and oxide layer formation on the modified surfaces led to reduced ion release, which contributed to improved corrosion resistance. Finally, the effect of the formed surface patterns on antibiofilm formation was investigated via incubation with *C. albicans* for 24 h, which exhibited that microdeformation patterns remarkably inhibited the biofilm formation. Throughout the experiments, certain patterns yielded outstanding results among the four patterns formed. Overall, it was concluded that forming controlled patterns on stainless steel surfaces via surface microdeformation significantly contributed to the metal’s biocompatibility via improving bioactivity, corrosion resistance, and antibiofilm formation properties. Especially, the specific surface properties such as increased surface energy, high surface roughness, and dislocation density close to the metal surface as well as increased hydrophilicity obtained via forming the pattern with relatively deeper and narrowly spaced indents yielded the most promising outcomes. These methodologies constitute novel approaches to be used while designing new methodologies for the surface modification of metallic implant materials for improved osseointegration.

## 1 Introduction

Metallic materials are widely used as biomaterials for various biomedical purposes such as orthopedic implants, dental applications, or cardiovascular devices.^1−6^ Biomedical alloys are especially preferred for orthopedic applications such as hip and knee prostheses as well as dental implants.^7−12^ Although lighter material alternatives have been developed over the years, the main reason biomedical alloys such as stainless steel, cobalt–chromium (Co–Cr), or titanium (Ti) based alloys are still preferred is their mechanical properties. For such load-bearing applications, metallic biomaterials provide the advantages of mechanical durability, fatigue resistance^12−17^ as well as the ease of adjusting mechanical properties by altering their microstructure.^4,14,18−20^ Moreover, these alloys also provide good biocompatibility, especially due to their improved corrosion resistance. However, for orthopedic applications, osseointegration, which is defined as the functional biomechanical fixation of the implant with the bone tissue, becomes another crucial factor affecting the biocompatibility and thus the success of the implant.^18,21,22^

Whether the orthopedic implant is biodegradable or not, its surface needs to show bioactive properties for tight bonding with the bone tissue and ensure osseointegration.^23^ Metal biocompatibility, osseointegration, and corrosion resistance can be improved by promoting the formation of bioactive hydroxyapatite (HA) and protective (oxide) layers.^9,11,23−28^ The tendency of calcium-phosphate (CaP) rich particle formation on metal surfaces is an indication of bioactivity which determines the bone formation ability on the implant surface.^18,20,21,23,28−32^ While evaluating the bioactivity of a material, immersion in simulated body fluid (SBF) methodologies are often used. In addition to the bioactivity evaluation, immersion in SBF can also be used to monitor deposition of bone–like apatite structures on the metal surface.^2^ Moreover, interactions with implanted prostheses and human body fluids also could be investigated via immersion in SBF. In this manner literature states that, the surface treatments have great impacts on the material bioactivity and cellular biocompatibility behaviors in terms of determining precipitation of CaP particles.^12,27,33−35^ Calcium-phosphate deposition could be triggered by various surface modifications. Deposited calcium-phosphate is then converted to bone-like apatite layer, promoting the bone tissue-material fixation ability and thus bioactivity.^13,18−20,22,23,34^ Therefore, as a result of well-characterized surface treatments on metal surfaces, bioactivity and biocompatibility could be enhanced through deposition of apatite-like molecules.^36,37^

In addition to bioactivity, other critical issues determining the success of osseointegration are the prevention of fibrous tissue growth and biofilm formation.^21^ Especially for prostheses and implants, microorganism colonization leading to biofilm formation on the biomaterial surface may delay or impede biomechanical fixation of the implant with the bone tissue and cause infections.^38−41^ Among the various microorganisms, *Candida* species are one of the most common fungi-associated microorganisms, causing biofilm-related infections. Studies stated that 95% of the infections that have occurred in the last two-three centuries are caused by *Candida* species.^42^ This high rate is also correlated with the infections caused by *Candida* species related biofilm formations as a result of the increasing use of implants and medical devices in the last century.^39,43^ According to National Institutes of Health (NIH) data, this rate constitutes 70–80% of total infections observed in humans.^40,42^ The formation of a biofilm belonging to the *Candida* species on implanted material surfaces in the body also increases the risk of invasiveness of the infection to other tissues and organs through the blood-flow.^41^ Vascular-catheter infections which also arise frequently due to *Candida albicans* constitute the fourth of the total *Candida* species related biofilm infections observed on biomaterials such as prostheses (e.g., hip, knee, and shoulder) as well prosthetic heart components.^40−44^ While they are commonly observed, *Candida* infections are also difficult to prevent due to their drug resistance and the natural persistence of fungi specifics.^40,42−44^ For this reason, it is of utmost importance to prevent *Candida* species related infections by manipulating the material surface properties in a way that will reduce biofilm formation, or if possible completely prevent, and by ensuring that the surface has antifungal properties. Thus, implant lifetime and patient comfort can be improved, while high cost medical operations which arise from biofilm-related infections and implant replacements can also be prevented.^38−40,44−46^

Since the first biomaterial-tissue interaction and thus the initial adhesion of the cells and microorganisms take place on the biomaterial surface, surface properties of the biomaterial are of utmost importance.^16,18,21,33^ Surface properties determining the biocompatibility and bioactivity response include parameters such as roughness,^12,13,21^ specifics of topographical features,^18,35,47^ wettability,^6,48,49^ and surface energy.^6,35,36^ Among these, the most commonly investigated parameters are the effect of surface roughness and its correlation with surface energy and wettability. Increased surface roughness, which is a measure of the deviations caused by surface irregularities forming the surface pattern and topography, is known to also increase surface energy.^3,4,6^ However, as opposed to the general understanding that surface roughness and wettability are directly correlated with each other, recent studies have shown that organization of the topographical features are also very effective in terms of determining wettability, initial adhesion and therefore biocompatibility of the biomaterial.^16,50,51^ Specifically, recent studies focusing on the effects of surface feature specifics on cellular behavior have shown that shape, size, depth, and repeating frequency of the units forming the surface topography strongly determine initial cell attachment as well as proliferation and differentiation behavior.^2,33,51^

Surface properties can be manipulated by various physical methods such as laser, plasma, and ion beam processing, sandblasting, polishing, and chemical methods (e.g., electrochemical passivation and anodization).^25,52^ The physical and chemical surface treatments create patterned surfaces that increase the surface roughness, topography, wettability, energy, and dislocation density of the material.^9,22,27,35,49,53−55^ Literature studies show that the properties of these manipulated surfaces, enable osteoblast cells to mineralize more easily,^31^ support cell–implant interaction,^9,16,35,56^ increase protein adhesion, provide better adhesion surface for cells, and therefore, promote the cellular proliferation, spreading, and differentiation.^16,24,27,35,47,57−59^ It is stated that surfaces containing micro/nanogrooves or channels have increased surface energy, roughness, wettability, also, certain patterns allow cells to attach better.^27,31,60^ In addition to the improvements mentioned above, manipulation of surface properties, also supports the surface bioactivity which leads to formation of calcium-phosphate, apatite and oxide layers on the material surface therefore improving osseointegration.^33,35,52^ Hydroxyapatite (HA) and other Ca–P rich apatite particles which are deposited on the material surface have a similar chemical composition to bone.^26,53,59,61,62^ In this way, it promotes fixation between implant and bone;^10,47,61,63,64^ therefore, it has been reported that increased success in solving fixation problems encountered in metallic implants, accelerated bone tissue formation,^47,59^ and also decreased elastic mismatch can be achieved.^2^ Modifying the surface properties and topography also affects the formation and stability of the natural protective layers such as the passive oxide layer. The passive oxide layer is related to the elemental composition and properties of the material. It repairs itself in the presence of oxygen, reduces the ion leakage,^10−12,20^ and can be localized on sites close to dislocation networks.^14,32,50^ The high iron (Fe), chromium (Cr), and nickel (Ni) content (up to 25%) of stainless steel supports the repassivation of the surface oxide. It is known that the oxide layer obtained on the stainless steel material surface reduces the ion release in corrosive environments such as body fluids as compared to the uncoated surfaces.^5,12,20,55^ Thus, biocompatibility and corrosion resistance are also increased,^14^ as the oxide layer and apatite deposition make the material surface more bioactive.^24^

While the aforementioned studies show that modifying the surface properties improve biocompatibility by promoting bioactivity and resisting biofilm formation, some of the recent studies have shown that, microstructural properties of metallic materials can also be critical for determining their biocompatibility at different levels.^22,65^ The common approach when investigating the microstructural mechanisms of metallic materials is focusing on the microstructure-mechanical property relationship. The linear defects in the microstructure of metals, which are named dislocations, enable the deformation of metals and determine the deformation behavior and mechanical properties of metallic materials. However, according to recent studies, the presence, density, and localization of dislocations in the metal microstructure also affect the biocompatibility.^2,50,51^ Specifically, studies investigating the microstructure-biocompatibility relations have revealed that, applications of controlled deformation to metals trigger dislocation activity close to the metal surface which influences surface topography and surface energy, and as a result, affect the biocompatibility at various levels.^1,5,6,51,55^ The biocompatibility responses to surface processing and resulting microstructural mechanisms explored so far include cell attachment, proliferation, and differentiation behavior as well as *ex situ* biocompatibility response in simulated body fluid environment.^1,3^ However, to the best of the authors’ knowledge, studies focusing on the effects of controlled surface patterning and microstructural mechanisms on the bioactivity and biofilm formation response of metallic biomaterials have not been reported yet.

With this motivation, the current study aims to investigate the effects of controlled surface micromodification and resulting surface properties as well as microstructural activities on the bioactivity, ion release behavior, and antibiofilm formation properties of metallic biomaterials. For this purpose, controlled microdeformation areas were formed on 316L stainless steel surface, composed of indents with varying sizes, repeating frequencies, and therefore different dislocation densities. The effects of resulting surface properties and microstructural mechanisms on the CaP formation and ion release behaviors as well as attachment and viability of *Candida albicans* were explored in detail.

## 2 Materials and Methods

### 2.1 Sample Preparation

Stainless steel (316L grade, Panchemical Steel Ltd.) was supplied as a cylindrical rod of 5 mm diameter. A total of 45 samples were obtained by cutting the rod into 3 mm-height pieces via wire erosion. Specimens were metallographically prepared via grinding with waterproof SiC sandpapers of increased grid density from 120, 320, 600, 800, 1200, to 2000. Grinding was applied with the same directional moves on each sample for the purpose of obtaining similar surface properties for every sample. The elemental composition of the steel used in experiments is given in Table 1.

**Table 1:** Chemical Composition of the 316L Stainless Steel in wt %

Element	Carbon (C)	Manganese (Mn)	Phosphorus (P)	Sulfur (S)	Silicon (Si)
Weight percent (%)	0.027	1.760	0.042	0.029	0.270

Element	Chromium (Cr)	Nickel (Ni)	Molybdenum (Mo)	Copper (Cu)	Nitrogen (N)	Iron (Fe)
Weight percent (%)	16.800	10.120	2.120	0.380	0.080	68.372

In order to observe the effects of surface microdeformation on the bioactivity and antibiofilm formation properties of the 316L steel, microdeformation patterns with different parameters (varying forces and distances between indent centers) were applied on the sample surfaces. Pattern applications were performed as described by Bicer & Toker (2023)^51^ in a recent study. Specifically, Vicker’s microhardness (Future Tech., Vickers hardness tester, FV-800 model) was used as a new method to form patterns consisting of 10 rows × 10 columns of indents which constitute the controlled microdeformation areas on the surfaces. According to this procedure, four different microdeformation patterns were formed and applied on the sample surfaces, as listed in Table 2.

**Table 2:** Different Parameters Used during the Formation of Different Microdeformation Patterns

Patterns	Application force (kgF)	Distance between indent centers (μm)	Exposure time (s)
**Pattern 1**	–	–	–
**Pattern 2**	1 kgF	100 μm	5 s
**Pattern 3**	1 kgF	200 μm	5 s
**Pattern 4**	0.5 kgF	100 μm	5 s
**Pattern 5**	0.5 kgF	200 μm	5 s

Pattern 1 was used as the control group. Nine samples were prepared for each pattern. Specimens were cleaned in an ultrasound bath in ethanol (70%, for 3 min) prior to the experiments. Before the static immersion and antibiofilm tests, subsequent to ultrasonic cleaning, samples were also autoclaved (Conditions; using steam, 121 °C for 15 min, 134 °C for 3.5 min).

### 2.2 Surface Characterization

Surface roughness parameters were obtained by profilometric measurements. Mitutoyo SJ-410 mechanical contact needle profilometer was used to measure average roughness (R_a_) and maximum indent depths (R_t_). During the profilometer measurements, it was made sure that measurement routes passed through the indent centers in order to obtain more consistent results. Additionally, measurements were taken in different directions on the samples of each pattern.

For preliminary microscopic characterizations, the microdeformation patterns were examined under Field Emission Scanning Electron Microscopy (FE-SEM)(Hitachi Regulus 8230 Field Emission Scanning Electron Microscope), in order to examine the pattern-related specifications such as indent size and repeating frequencies.

In order to further investigate how dislocation activities are induced via the microdeformation process and observe the various dislocation densities on the surfaces with different patterning parameters, the optical microscope equipped with the Vickers microhardness tester used for mirodeformation (Future Tech. Vickers Hardness Tester FV-800) was utilized. An optimum magnification of 200× was selected for visualization.

### 2.3 Contact Angle Measurements

Contact angle measurements were conducted via Attension Theta Optical Tensiometer (Biolin Scientific, Gothenburg, Sweden) with the use of a sessile drop technique which involves dropping deionized water onto the sample surfaces. The samples were cleaned ultrasonically for 15 min in ethanol (70%), then acetone (99.5%) prior to the measurements. Measurements were taken using 2 μL of deionized water over microdeformed areas. Water drop images were taken with a high-speed camera. Average contact angle values were obtained by taking measurements on three samples for each pattern, while for each sample, an average of the right and left angle values were taken.

### 2.4 Static Immersion Test

The samples were subjected to static immersion in a densified simulated body fluid (3X-SBF). The purpose of applying the static immersion tests in a densified SBF environment was to trigger CaP deposition in order to be able to compare the CaP deposition tendencies of the different patterns and thus the bioactivity on the different surfaces in a shorter time span. Concentrated SBF was prepared according to the Kokubo protocol, by adding the listed chemicals step by step (Table 3) into 700 mL distilled water. A polypropylene beaker was selected, and during preparation, the temperature was set at 37 °C, and stirring was applied at 250 rpm to prevent chemical precipitation. Finally, pH was set at 7.4 after distilled water was added until a 1 L volume is completed.

**Table 3:** Chemical Composition of the Densified Simulated Body Fluid (3X-SBF)

Chemical adding step	Chemical	Amount in grams/Liter
**1**	NaCl	23.988 g
**2**	NaHCO_3_	1.05 g
**3**	KCl	0.732 g
**4**	K_2_HPO_4_.3H_2_O	0.684 g
**5**	MgCl_2_.6H_2_O	0.915 g
**6**	1M–HCl	120 mL
**7**	CaCl_2_	0.834 g
**8**	Na_2_SO_4_	0.213 g
**9**	(CH_2_OH)3CNH_2_	18.171 g

The samples were subjected to static immersion in 3X-SBF for three different time periods (2, 4, and 6-days), and for each immersion period, three sets of samples were used. According to the Kokubo protocol, the sample area to SBF volume ratio was set at 1/10, where each sample was subjected to 8.64 mL of SBF (since the sample area was 86.4 mm^2^). SBF-added samples were kept in a temperature-controlled water bath to maintain the body temperature of 37 °C until the end of each time period. 3X-SBF solutions were renewed with freshly prepared 3X-SBF every 2 days for each immersion period. At the end of the three immersion periods, the samples were retrieved from the solutions and examined in detail visually by FE-SEM and elementally by energy-dispersive X-ray spectroscopy (EDS). The reason EDS was preferred for the characterization of deposited particles was to be able to detect and identify the particles which were specifically located in the close vicinity of the indents and therefore distinguish between the effects of the different surfaces on deposition of CaP rich particles.

In order to confirm whether the deposited CaP rich particles are hydroxyapatite (HA) or not, the samples which were subjected to the 3X-SBF environment for the longest immersion period (6 days) were also analyzed via X-ray diffraction (XRD) (Panalytical Empyrian). The XRD analysis was conducted at a 2θ angle range from 20° to 40°.

For thoroughly understanding how the chemical changes on the samples with the different surface properties affect ion release from the samples into the SBF solution, inductively coupled plasma mass spectrometry (ICP-MS) analysis was conducted, which is a good indicator of the protectiveness of the surfaces against ion release since it provides a comparison among the protectiveness of the CaP layers formed on the different surfaces and thus the different surface properties. For this purpose, the retrieved CaP deposited samples were immersed in 1X-SBF (which was prepared with the sample protocol and the chemicals listed in Table 3, where the amount of each chemical was divided by three) to evaluate the long-term ion release behaviors. According to the initial SEM observations, 6 days of immersion in 3X-SBF yielded the most intense CaP deposition. Therefore, they were chosen for long-term immersion tests to analyze the protectiveness of the formed CaP layers on the different surfaces in terms of ion release, since CaP layers expected to impede ion release by behaving as a protective layer.^2,3,18,23,55,60^ CaP deposited samples were immersed in 1X-SBF with the same sample area to volume ratio of 1/10, in separate tubes with covered lids, and placed in the same temperature-controlled water bath where the body temperature of 37 °C was maintained for 30-days. At the end of the 30-days immersion period, SBF solutions were collected from each sample for the examination of their ion release rates from each sample via ICP-MS. Considering the elemental content of 316L stainless steel (Table 1), iron (Fe), nickel (Ni), copper (Cu), chromium (Cr) and molybdenum (Mo) elements were selected to evaluate for the potential ion release amounts from the samples with different surface patterns, which were expected to affect the CaP deposition amount on each sample. The samples were also weighed prior to and following the 30 days of immersion in 1X-SBF (after drying), in order to evaluate the differences in their weight which may stem from the deposition of apatite structures and the released ion amount over time.

### 2.5 Biofilm Formation and Characterization

The yeast *Candida albicans* (ATCC 14053) was selected to examine the biofilm formation mechanisms due to being a part of the human microbiota which frequently causes biofilm formation on implant surfaces.^42,67^ Cells were inoculated on the metal surfaces (Mc Farland is 0.5; 1–5 × 10^6^ CFU/ml) and incubated at 37 °C for 24 h (in RPMI 1640 medium). In order to observe the biofilm formation, cells were fixed on the sample surfaces in a 1% glutaraldehyde fixative solution for 24 h. Then, samples were rinsed with PBS for three times. Dehydration was conducted by applying gradient series of ethanol (30%, 50%, 70% and 96% ethanol). The adhesion behaviors of the cells on 316L stainless steel sample surfaces were then characterized via scanning electron microscopy (FE-SEM, Hitachi Regulus 8230).

### 2.6 Cell Counting

The yeast cell densities per unit area were calculated from the SEM images of the samples which were obtained after 24 h of incubation via ImageJ program and Python software. ImageJ was used to measure the cell density on the control sample, while Python was preferred for the microdeformation applied sample surfaces, as the microdeformation indents caused complications in terms of contrast during the cell counting step. On the patterned surfaces, first binary images were obtained to measure cell densities from the SEM images of the microdeformation applied samples. High detection accuracy was achieved by manually selecting the cells that could not be identified as cells or could not be detected. White-black pixel ratios were adjusted by using image processing principles on binary images within the python program. In this way, the cell-covered area/total surface area in the examined region was calculated. Cell densities were then normalized for the microdeformation applied samples with the use of data, which are the number of cells per mm^2^ that was obtained for the control sample via image J program and the cell density ratios obtained from python software. Measurements were taken from 5 different regions for each pattern, so average cell densities per unit area and standard deviation outcomes were obtained.

### 2.7 Statistical Calculations

Minitab 19 was used to statistically evaluate the data. Correlated measurements were tested via a one-way ANOVA statistical method at 95% confidence interval. Subsequently, the Tukey pairwise comparisons were used to determine the significance of differences obtained among the patterns.

## 3 Results and Discussion

**Figure 1:**
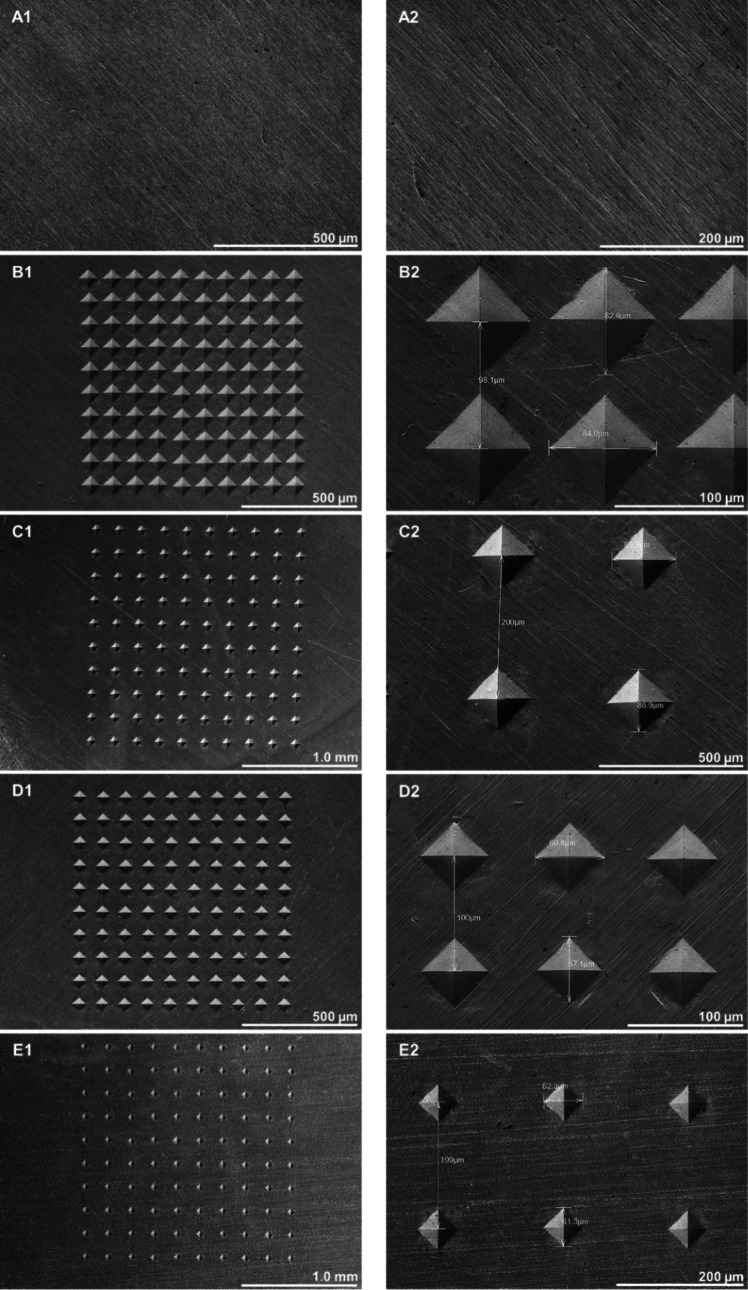
FE–SEM images of patterned and control samples. The images on the first column include the total of the patterned areas, second column shows indent dimensions and distances between the indents under higher magnification. (A1–2: Control/Pattern 1, B1–2: Pattern 2, C1–2: Pattern 3, D1–2: Pattern 4, E1–2: Pattern 5 sample).

For the preliminary structural examination, the microdeformation applied and control sample surfaces were investigated via FE-SEM. The SEM images were initially taken at 45× magnification for all of the sample groups to include the whole pattern on the microdeformation applied samples, which was followed by higher magnification imaging, where pattern details of each of the microdeformed samples as well as the differences between the groups can be observed (Figure 1).

The images in Figure 1 showed that the patterning parameters were applied on the surfaces according to the priorly set parameter values, and the images also proved that surfaces do not include any unexpected structural and topographical properties. Moreover, the images taken under the same magnification show the relative indent sizes and how the indents are spaced for the patterns with different applied force parameters and different distance parameters between indents. Following the initial observation of the effects of various parameters on patterned sample surfaces via SEM, the sample surfaces were examined via profilometer to obtain detailed information about the surface and topographical properties.

**Figure 2:**
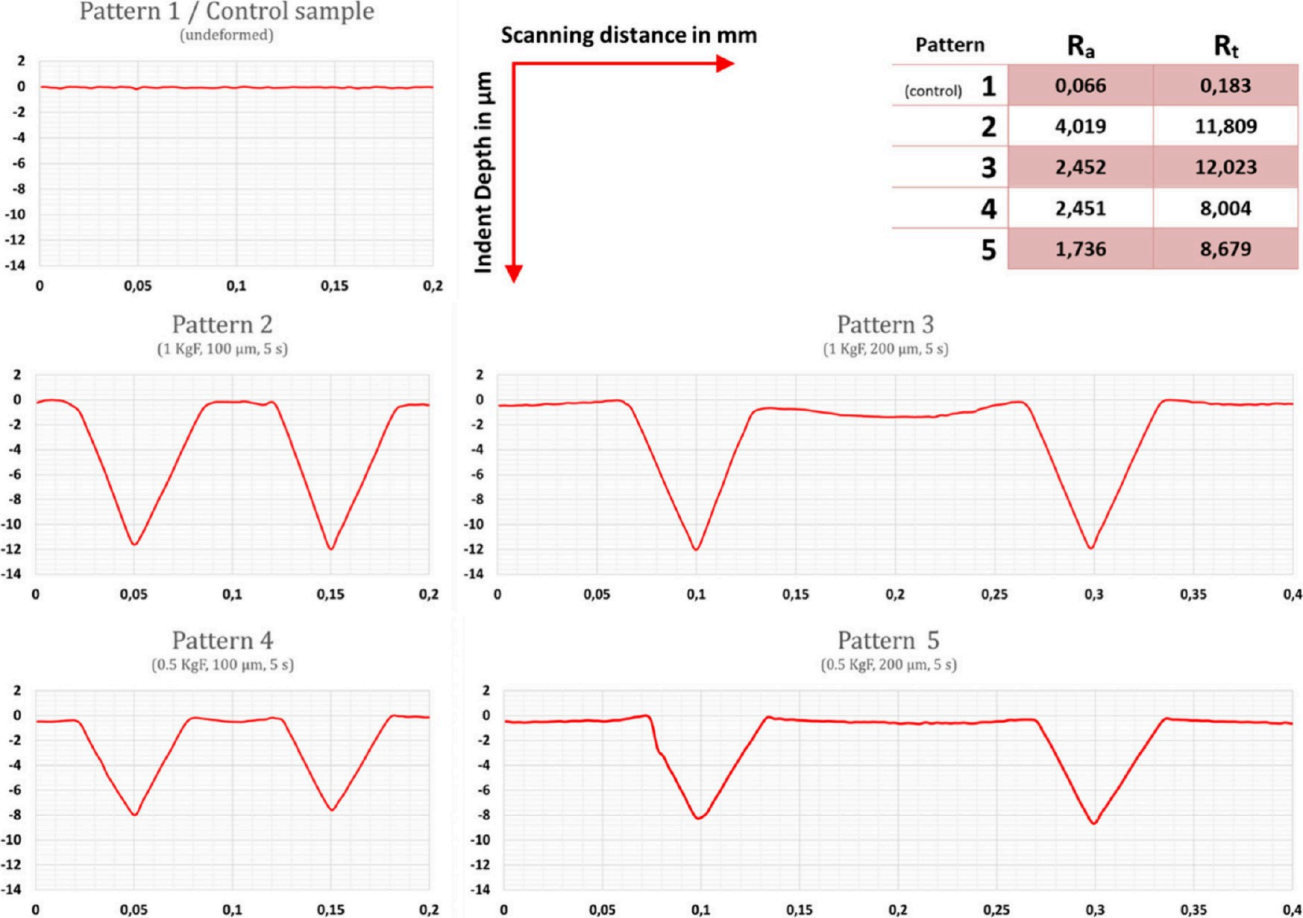
R-profiles and roughness measurements of the sample surfaces.

The measured surface roughness parameters which are average roughness (R_a_) and maximum depth of indent (R_t_) values, as well as the two-dimensional R profiles those belonging to each pattern is given in Figure 2. R-profiles were taken from the patterned area, and it was made sure that the measurements were taken so as to pass through indent centers in order to avoid deviations in roughness parameters, especially R_t_ values. Two indents were included in each R profile with the purpose of easy comparison between the patterns.

Findings of the roughness measurements exhibited that the lowest R_a_ and R_t_ values were obtained for the control sample surface. On the patterned surfaces, the main factors affecting the roughness parameters were the amount of the applied force and the distance between indents. Specifically, it was observed that applied force proportionally affects R_a_ and R_t_ so, the higher amount of applied force on patterns 2 and 3 resulted in relatively high R_a_ and R_t_ values on these surfaces. Moreover, the distance between indents is inversely proportional to R_a_ values. As the distance between the indents increased, R_a_ values reduced, nonetheless R_t_ values were not affected. According to the aforementioned information, the highest R_a_ and R_t_ values were obtained on pattern 2 and the lowest R_a_ and R_t_ values were obtained on pattern 5 among the patterned groups. While the R-profiles obtained for the samples patterned with the same force are similar, the distances between indents were found to cause slight differences in indent depths, although the same force was applied. Specifically, the samples patterned with a 200 μm distance between indents have slightly deeper indents as compared to the patterns with a 100 μm indent distance. The reason for this difference is the higher dislocation density, which forms as a result of the close spacing of the indents and therefore causes local strain hardening around the indents.

**Figure 3:**
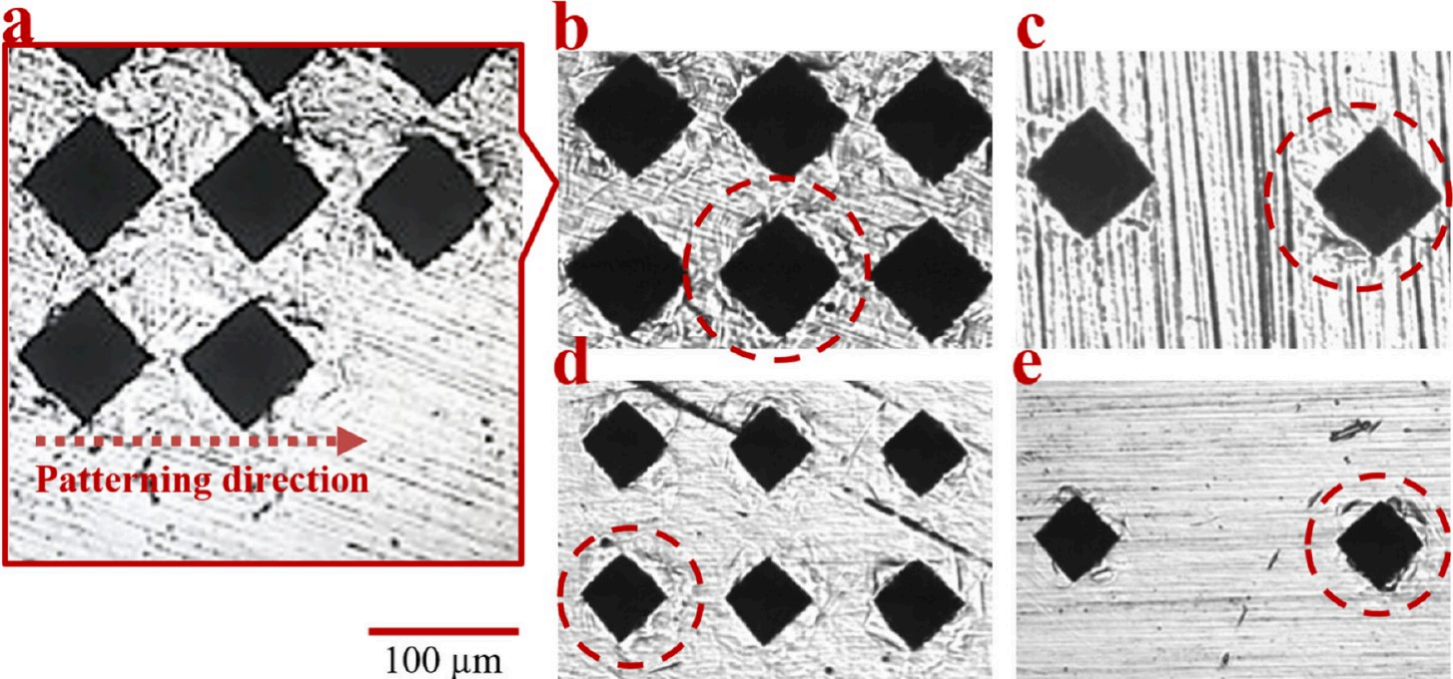
Optic microscope images exhibiting; a) dislocation formation during patterning via microdeformation process of, b) pattern 2, c) pattern 3, d) pattern 4, e) pattern 5, dotted circles representing dislocation densities around single indent.

For observing the dislocation activities triggered by the microdeformation process and comparing the effects of the different patterning parameters on microstructure close to the surface, namely, dislocation densities, optical microscopy images were used (Figure 3). The reason optical microscopy was preferred for this purpose was the ability to observe the formation of dislocations around the indents as the microdeformation procedure proceeded (Figure 3a). Specifically, from Figure 3a, it can clearly be observed that, while the area that has not yet been processed is clear, the patterned area exhibits a high density of dislocations, whose progression can be tracked along the patterning direction. This observation both proves the dislocation activity triggering mechanism of the microdeformation procedure, and the effect of indent depth and spacing on dislocation density.

**Figure 4:**
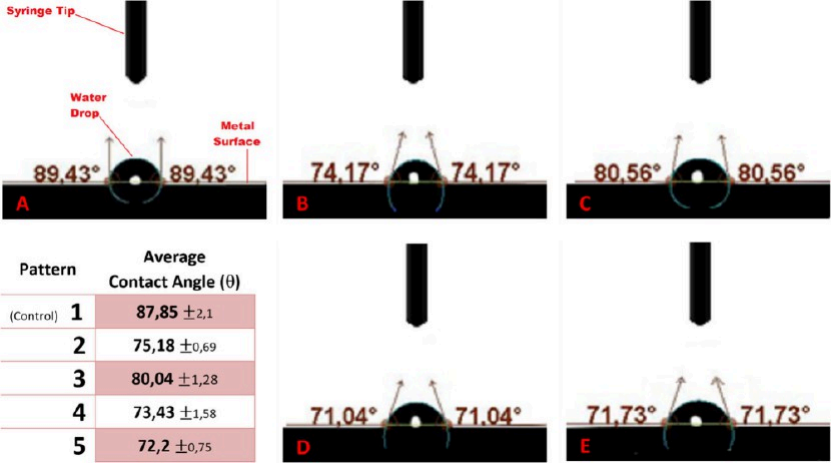
Contact angle measurements of the surfaces. A: Control/Pattern 1, B: Pattern 2, C: Pattern 3, D: Pattern 4, E: Pattern 5.

In a similar study where microdeformation was applied on 316L stainless steel surfaces, Bicer Toker (2023)^51^ showed that dislocation activities increased as the indent depth increases, and as the indents are more narrowly spaced. Similar to these findings, the most intense dislocation activity is observed for pattern 2 while the lowest dislocation density is observed on pattern 5 (Figure 3). As dislocations are high energy microstructural features, the surfaces with higher dislocation densities are also expected to exhibit surface higher energy levels. Therefore, the triggered dislocation activities are also anticipated to tend to reduce the contact angle by increasing the surface energy, thereby increasing the hydrophilicity of the surface.^51^ Contact angle measurements are important for determining the hydrophilicity or hydrophobicity of microdeformed sample surfaces. Determining the contact angle and wettability characteristics of a metal surface is critical for a thorough understanding of the mechanisms of CaP precipitation and biofilm formation on modified surfaces. For this reason, contact angle measurements were carried out in order to better understand the effects of microdeformation, triggered dislocation mechanisms on cellular activities, and bioactivity of the implant material. The average contact angle values of the samples are given in the Figure 4 with the measurement photos.

Among the contact angle measurements, the highest average contact angle value was measured on the control group, which is the group with no microdeformation application. It was determined that the contact angle values decreased on all the microdeformation applied samples compared to the control group. According to the decrease in contact angle, wettability and hydrophilicity properties of the sample surfaces were enhanced. These properties were mainly affected by the microdeformation parameters. Surface energies of the microdeformed samples were expected to increase as a consequence of the triggered dislocation densities.^51^ Moreover, roughness is another parameter that causes an increase in the surface energy as well as hydrophilicity. Therefore, the decrease in contact angles and so the increasing hydrophilicity of the samples with microdeformation can be attributed to the enhanced surface energies of these surfaces due to the increased roughness and triggered dislocation activity close to the surface^51,66^ In the former studies, there are findings that wettability can be also affected by geometry specifics of the surface topographical features, especially by the movement of the measurement liquids into the deep grooves which show that detailed investigations are required to make correct assumptions.^66^Figure 4 exhibits that wettability properties of the surfaces with microdeformation were also more dominantly affected by the depth of the indents rather than surface roughness and surface energy. The dislocation density of the microdeformed surfaces increases with indent depth and diminishes as the distances between indents get wider (Figure 3) and surfaces with higher dislocation densities are expected to have higher wettability due to increased surface energy.^51^ According to the aforementioned information, lower contact angles and thus higher wettability were expected on patterns 2 and 3 as compared to patterns 4 and 5; however, patterns 4 and 5 exhibited higher wettability. Former studies also evidence that as the depth of the surface grooves increases, air is more likely to be trapped within the groove, acting like a barrier against the penetration of the liquid through the groove and thus exhibits lower wettability. Such surfaces are considered to behave like a composite material due to the air trapping within their indents. This case is explained as the Cassie–Bexter model in the literature.^67^ The surfaces exhibiting the Cassie–Bexter model resist the penetration of the fluids into air-trapped indents therefore become more hydrophobic.^67^ This phenomenon can also be used to explain the lower hydrophilicity of patterns 2 and 3 in the current study, which exhibit higher surface energy yet also consist of deeper indents which resist wettability according to the mechanism explained with the Cassie–Bexter model.

**Figure 5:**
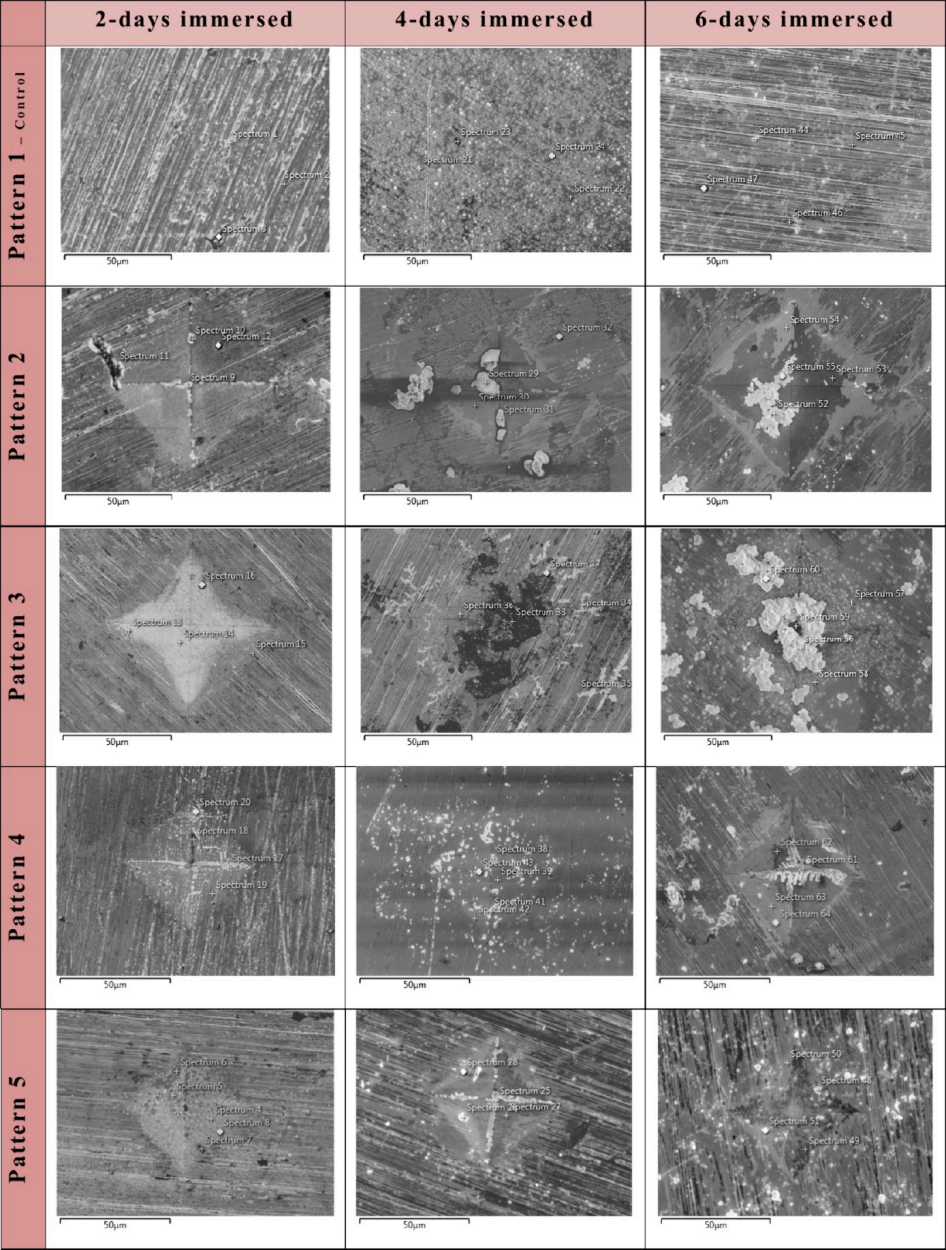
FE-SEM images from sample surfaces following immersion in densified 3X-SBF for 2, 4, and 6 days (each image was obtained at 1000× magnification). (Spectrum marks show the spots where the EDS readings were taken).

Surface roughness parameters can be changed along with the creation of microdeformation patterns on sample surfaces, additionally it is known that the surface energy and dislocation activities of the material can be triggered in this way.^4^ Various studies with similar metallic biomaterials have shown that surface energy increases when samples are plastically deformed.^4,6^ Thus, it has been seen that the deposition of apatite-like structures is supported along with increased energy and dislocation activities on such modified surfaces.^36,55^ Since in the current study it is aimed to modify the stainless steel surface into a more bioactive orthopedic implant surface which can directly contact with the bone tissue, the formation of apatite-like structures on the modified metal surface was also investigated in detail. For this purpose, the samples were immersed in densified (3X) SBF and the material surfaces were examined by FE-SEM following incubation periods of 2, 4, and 6 days. In order to understand the effects of different microdeformation patterns on CaP deposition and thus bioactivity, the images were obtained as close as possible to the indents. SEM images of CaP deposition behaviors on the different patterns following immersion in 3X-SBF for periods of 2, 4, and 6 days are given in Figure 5.

According to the obtained SEM images, microdeformation applied surfaces promoted deposition of a higher amount of precipitates as compared to the control sample surface. Particle depositon on the control sample surface was significantly lower than microdeformation applied surfaces, and there was no increase in precipitate amount over time. This observation is in agreement with a similar study, where more calcium-phosphate (CaP) formation was obtained on modification applied surfaces since higher energy surfaces are expected to promote new particle deposition.^9,64^ Evaluating the deposited particle characteristics, 2-days immersion results showed that smaller precipitates are evident on the control sample and the samples patterned with 0.5 kgF (patterns 4 and 5), while larger precipitates were obtained on samples patterned with 1 kgF (patterns 2 and 3). This result can be attributed to the higher R_a_ and R_t_ values of the patterns 2 and 3. Similarly previous studies also show that, after modifying the stainless steel and titanium alloy surfaces with laser, better calcium-phosphate and hydroxyapatite adhesion were obtained on the material surfaces with higher surface roughness.^9,31,47,68^ It can be suggested that surfaces with higher surface energy, more intense dislocation activity, and deeper indents provided a more suitable environment for the deposition of precipitates. In studies conducted by various researchers with metallic biomaterials, it has been indicated that high-energy surfaces are important for the deposition of biological molecules.^5,18,48,60^ Similarly, it is possible to make similar comments for the 4-days results. It was observed that larger and denser precipitates deposited on the microdeformed surfaces, especially on the samples patterned with 1 kgF, and with the extended immersion duration, the precipitates started to form connections with each other. On the samples patterned with 0.5 kgF, it is seen that there are fewer precipitates and less interaction between the precipitates, and the precipitates are mostly located within the indents, especially on the diagonal lines of indents. 6-days results also supported these observations. When the results were evaluated as a whole, it was observed that higher roughness (R_a_) and increased indent depths (R_t_) supported the formation of precipitates on the microdeformed samples, which are in agreement with the findings of former studies.^47^ The higher surface energy and dislocation density depending on the surface topographical properties supported the formation of precipitates.^68^ When the results of the samples patterned with 1 kgF and the samples patterned with 0.5 kgF were compared, the deposition of a higher amount of precipitates on the surfaces patterned with 1 kgF (patterns 2 and 3) showed the importance of dislocation density, surface energy, surface roughness, and indent depth. It can also be suggested that the distance between the indents was less effective on new particle deposition; however, since increased distance between indents reduces the dislocation density, it is also effective in terms of inhibiting the interactions of precipitates in between indents. Therefore, the early and higher amount of precipitate deposition on patterns 2 and 3 can also be attributed to the higher dislocation density of these patterns, which also trigger new particle precipitation.

**Figure 6:**
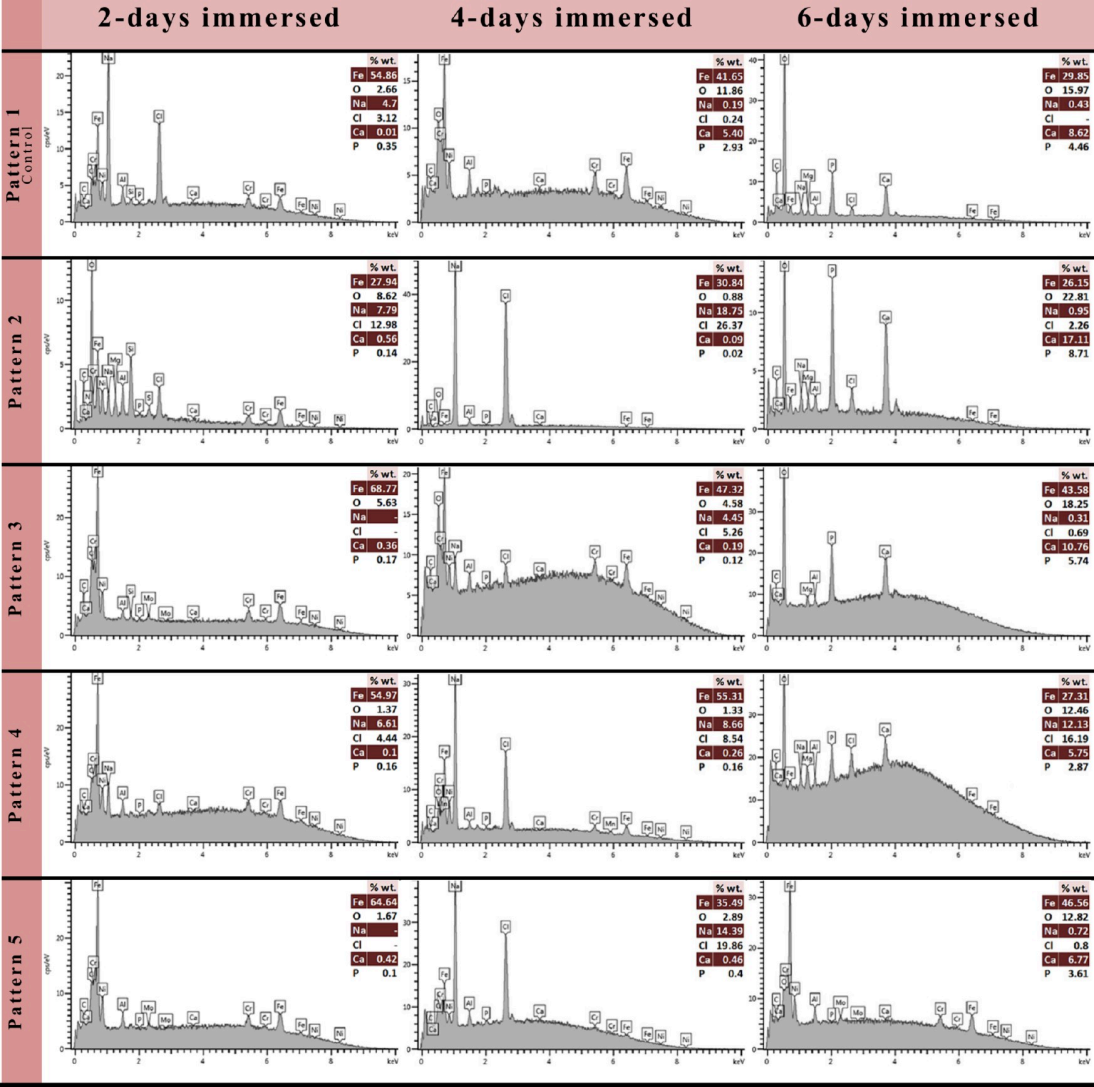
Results of EDS analyses with spectra taken from the areas close to the indents. (The tables on the right handside of each spectrum represent the average values of the signals from spesific indicator elements of interest.)

In order to obtain detailed information about the chemical content of the precipitates accumulated on the material surfaces, EDS analysis was also carried out. The results of the EDS analyses are given in Figure 6.

EDS analysis results obtained from various spots on the FE-SEM images are given in Figure 6. When the microdeformed samples were examined, salt precursors such as sodium (Na^+^) and chlorine (Cl^–^) were frequently encountered in the results of 2 and 4 days of immersion. In the 6-day immersion results, in addition to salt precursors, high amounts of Ca and P elements were also detected. It is known that salt precipitates or their precursors are early markers indicating the capability of formation of apatite-like structures. Specifically, it has been reported by previous studies that, accumulation of Na and Cl ions on the metal surface makes these surfaces adequate for interaction of tissue-specific proteins and better attachment of bone cells,^63^ and the hydroxyapatite deposition increases by the salt concentration.^62^ The increased calcium-phosphate (CaP) deposition on the 6 days of immersion in the current findings confirm these information.

It is known that hydroxyapatite (HA) structures on the metal surface shorten the healing time^21,37^ and reduce ion release in the body fluid environment.^22,32^ Therefore, in order to ensure an effective interaction between the biomaterial and bone-tissue, formation of an apatite-like bioactive layer on the biomaterial surface is essential.^13,53^ Calcium (Ca^2+^), phosphorus (P^3–^) and oxygen (O^2–^) elements are abundantly included in the structure of HA^19,21,25,62^ or other types of CaP.^53^ Calcium-phosphates are formed by the interaction of calcium and phosphorus elements, which are then converted into bone-like crystal apatite.^18^ Calcium-phosphates are known to support osteogenesis and angiogenesis,^13,30^ so these structures are important for improving the material’s biocompatibility and bioactivity.^33^ The presence of Ca, P and O elements evidence the initial formation of protective layers such as the oxide layer, as well as the deposition of CaP-type formations or hydroxyapatite.^18^ Therefore, since Ca, P and O elements are indicative of apatite-like bioactive layer formation, EDS readings were mainly focused on the analysis of those elements. The observations yielded that amounts of the Ca, P and O elements increased on all the sample surfaces with increasing immersion time. However, apatite-like structures were rarely encountered on the surface of sample 1. The reason why less deposition occurred was correlated to lower surface energy, dislocation density, and topographic properties (e.g., R_a_ and R_t_). From the past studies, it is known that the increased surface roughness and the resulting increased surface energy results in more favorable surfaces for the formation of such structures.^5,17,24,34,36^ It has been stated that CaP formation stages are primarily ion adsorption, then nucleation of nanosized particle formation, followed by lateral fusion of crystal^53^ and after CaP transforms into stable crystalline bone-like apatite.^20^ The initial presence of Ca ions on the surface provides a more favorable environment for HA formation,^62^ and the increase in Ca amount indicates the formation of chemical bonds between the metal surface and deposition of CaP.^30^ Among the tested surfaces, pattern 2 was the surface that exhibited these steps for the formation of a bone-like apatite structure with the structure and the amount of Ca which increased over time during the immersion periods.

In the literature, it was stated that the ideal Ca/P ratio resembling the HA formation is 1.67, and for a successful deposition of HA and CaP, the measured ratio should be 1.67 or higher. This ratio varies in structures such as bone, dentin and enamel as well as depending on age, gender and bone sites.^12,25,29^ It can be seen from the current EDS results that the Ca/P ratio is approximately 2 and even higher in the 6-days immersion results especially on the microdeformation applied surfaces. Another elemental ratio that gives the information about the bioactivity of precipitates is the Ca/Fe ratio that is correlated with CaP layer thickness in literature.^29^ Current EDS data showed that the Ca/Fe ratio increased significantly especially following 6 days of immersion. Moreover, the Ca/Fe ratios are much higher on the microdeformation applied surfaces compared to the control sample. Higher Ca/Fe ratios were obtained on pattern 2 at each immersion period among the pattern types.

**Figure 7:**
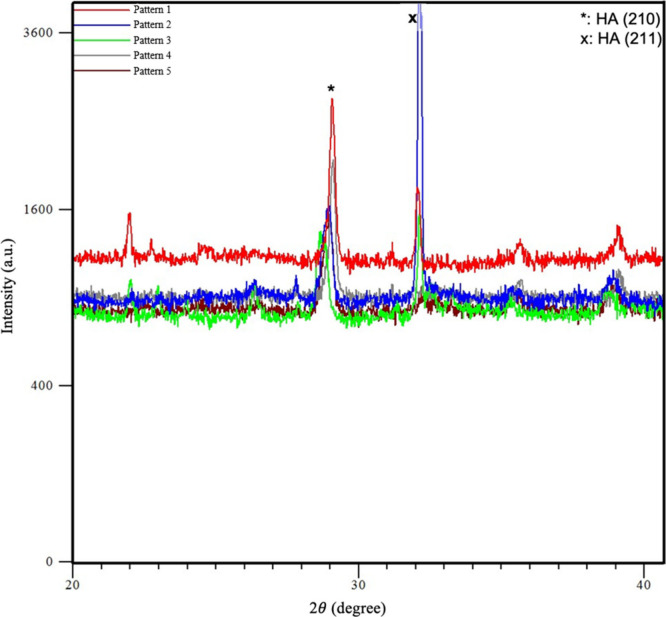
XRD patterns of the samples immersed in 3X-SBF for 6 days. (HA: Hydroxyapatite, the two predominant peaks matching the (210) and (211) diffraction planes of HA are marked.)

In order to confirm that the formed Ca–P rich particles are HA, the samples that were subjected to 3X-SBF for 6 days, which exhibited the highest amount of precipitates (Figure 5) and a much higher amount of CaP deposition (Figure 6), were also analyzed via XRD (Figure 7). The XRD patterns of all the samples matched that of HA, as the literature suggests that HA phase formations are likely to be observed predominantly in the 2θ range of 25–35° interval and two predominant peaks, namely those for the (210) and (211) diffraction planes of HA were detected in the XRD patterns of all samples, indicating the formation of HA in densified SBF environment for all samples.^21,22,29,37,69,71^ Moreover, the relatively stronger (211) peak observed in the XRD pattern of sample 2 also supports the findings that this specific microdeformation pattern provides a more favorable surface for the CaP-rich particle and HA deposition (Figure 7).

While the calcium and phosphorus signals observed on material surface after static immersion tests are indicators of the formation of apatite structures,^6,9,36,55^ the oxygen signal is a critical indicator of formation of the oxide layer.^64,70^ Accordingly, the abundance of oxygen on the sample surfaces following 6 days of immersion suggested that a rather apparent oxide layer has also formed on these surfaces in addition to the CaP layer and the naturally formed passive oxide layer. In SEM observations (Figure 5), a nonoxide layer containing surface is usually light gray, while, its color turns into dark gray with the formation of the oxide layer.^57^ In line with this information, when observed location wise, the oxide layers were generally located inside and/or around the microdeformation indents. Significantly among the samples, more dark gray areas (oxide layer) were obtained in pattern 2 samples. These results also suggest that surface microdeformation patterns promoted the new particle deposition, specifically, apatite and protective oxide layer formation. The fact that Ca, P, and O elements were observed in much higher amounts on patterns 2 and 3 as compared to the other samples evidence that these samples presented more favorable surfaces for the formation of hydroxyapatite in comparison to patterns 4 and 5. Moreover, surface properties of these samples were less favorable for the adhesion, and formation of precipitates on the high-energy indent regions as compared to patterns 2 and 3, as evident by the random and nonhomogeneous distribution of the precipitates on patterns 4 and 5. Therefore, these findings also show that the higher surface energy and dislocation activities caused by the topographic features on pattern 2 provided a more suitable environment for the formation and deposition of salt and apatite-like structures as well as a protective oxide layer in comparison to the other groups. Similar to the tendency of deposition of other precipitates on high energy surfaces, the areas with higher dislocation density due to the application of the microdeformation procedure are expected to induce oxide layer precipitation as well.^1^ Former studies also supported these findings, where the oxide layer formation preferentially occurs close to the dislocation areas.^1,3,65^ Furthermore, pattern 2 yielded a more advantageous surface for the interactions and homogeneous distribution of the precipitates as compared to pattern 3 as well, because of the closer distance between indents, which also contributed to dislocation density, surface energy, and roughness. In a similar study conducted with titanium, it was stated that bioactivity decreased with increasing spacing values between surface indents.^35^ Moreover, the immersion in 3X-SBF results of pattern 3 on the 2 and 4 days of immersion also showed that significantly fewer precipitates formed on its surface as compared to pattern 2. A combination of these evidence also exhibits that pattern 2 and its surface parameters are more suitable for the adhesion and deposition of CaP and oxide layer particles, which indicate bioactivity improvement. Therefore, the obtained results showed that microdeformation patterns are effective for material bioactivity, specifically with the parameters of pattern 2.^37^

**Figure 8:**
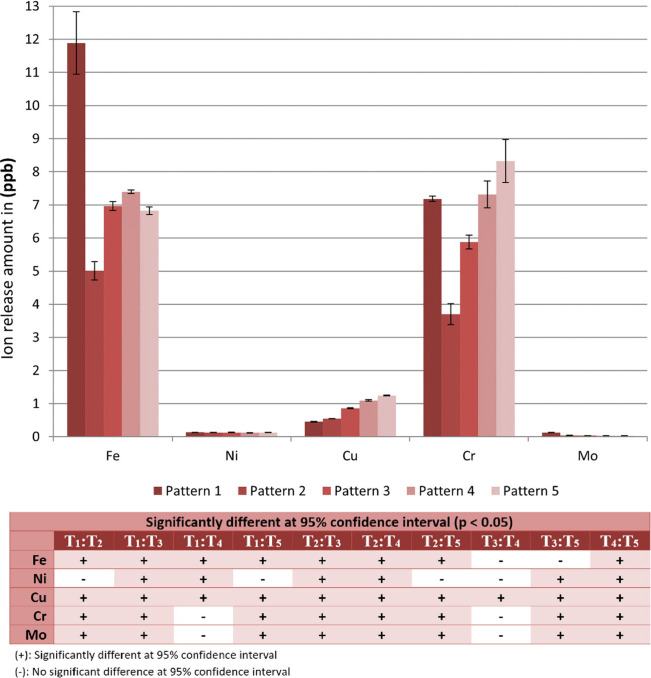
Average ion release amounts from the samples (previously immersed in 3X-SBF for 6 days) measured by ICP-MS after 30 days of static immersion in 1X-SBF. (Results were statistically evaluated via ANOVA and compared with Tukey test at 95% confidence interval (*p* < 0.05). The significant differences are represented within the table.)

Biocompatibility can be improved by promoting osseointegration^31^ which could be achieved by surface topographical manipulations.^35,48^ Literature studies put forward that rough and porous surfaces promoted cell adhesion and nucleation of secondary apatite as well as increased implant-tissue interaction, thereby supporting osseointegration.^9,21,59^ In addition, bone tissue formation can be improved by the presence of CaP structures on material surfaces since CaP precipitates are first deposited and contribute to the formation and maturation of bone minerals.^53^ Moreover, it was stated that CaP structures promoted the formation of protective oxide layer and provide resistance against ion release.^2,3,23,55,60^ Therefore, CaP formation on metal surfaces contribute to the biocompatibility of metallic implant surfaces by impeding ion release.^18^ Oxide layers formed in body fluid environments are in a continuous cycle of dissolution and reformation.^1,60,65^ In corrosive environments, such as body fluids, high surface energy might be expected to increase ion release. For this reason, the formation properties and protection of the oxide layer should be examined in relation with the ion release amounts depending on time.^60,61^ For this purpose, samples that were immersed in densified 3X-SBF medium for 6 days were preferred while investigating the ion release behavior, in order to understand the protectivity characteristics of the CaP and oxide layer formed on each sample. Therefore, these samples were also statically immersed in 1X-SBF for 30 days to evaluate the long-term ion release behaviors of the preliminarily CaP deposited surfaces. Following the 30-days of immersion, the metal ions that were released from the sample surfaces toward to SBF solutions were analyzed via ICP-MS. The potential release of metallic ions are known to cause toxicity, carcinogenicity, hypersensitivity, allergy, local tissue toxicity, osteolysis above certain threshold values.^11,16,72,73^ For this reason, released ion types and their amounts are crucial for implanted materials in the body,^10,58,72^ and these are also deterministic for the metal biocompatibility and bioactivity. The changes in the amounts of iron (Fe^2+^), nickel (Ni^2+^), copper (Cu^2+^), molybdenum (Mo^2+^), and chromium (Cr^2+^) elements, which are critical considering the elemental content of 316L steel, released into the SBF environment for each sample are given in Figure 8 as parts-per-billion (ppb) (namely, μg/L).

According to the literature, various studies state that threshold values change depending on age, and physical characteristics of the patients; which vary between 520 and 1750 μg/L for Fe, 120–500 μg/L for Ni, 417–1500 μg/L for Cu and 9–28 μg/L for Cr,^15,56,61,65,74^ Considering the ICP-MS data that shown in Figure 8, released ion amounts from the 316L stainless steel sample surfaces do not have any toxic, allergenic, genotoxicity, carcinogenic activities or undesirable effects depending on the literature thresholds. Additionally, ICP-MS findings supported the SEM, EDS results and literature. Taxell and Huuskonen (2022)^61^ similarly stated that, no carcinogenic, allergenic, genotoxicity or toxic effect was determined in a study of stainless steel implants placed in various parts of the body, which release very low amounts of metal ions (such as nickel, iron). The current results also evidence that the formation of microdeformation areas on the 316L stainless steel did not cause any negative effects in terms of ion release. Considering the chromium release amounts as a selective parameter for patterns, since the release rates obtained for the control sample as well as patterns 4 and 5 were around the limit value, it can be clearly seen that patterns 2 and 3 among the samples yielded improved resistance against ion release by the formation of a more protective layer through the chemical changes on their surfaces and thus promote biocompatibility. When ICP-MS and EDS results were taken into consideration and evaluated together, parallel to the promising EDS results of the current study, ion release results also show that fewer Fe and Cr ions were released from the samples with patterns 2 and 3 which evidence that the passive oxide layers and CaP precipitates formed on these patterns are more protective in terms of preventing ion release.^65,72^ Moreover, it can be deduced that the sample with microdeformation pattern 2 released fewer ions for each investigated element as compared to the other patterns. In a similar study it was stated that more ions were released in samples with low roughness values.^16,56^ It is known that the chromium (Cr) element contributes to the corrosion resistance of stainless steel by forming a passive oxide layer on the metal surface. The current results also show that Cr ion release is the lowest from the sample with pattern 2, which also evidence both that the passive oxide layer on this pattern is more stable^27^ and the CaP layer that formed on this surface is more protective against ion release.^10,21,72^

During the 30 days of immersion in 1X-SBF, the sample weights could also be affected, specifically can increase or decrease as a result of ion release or further deposition of particles. So, in order to evaluate these possible weight changes, samples were weighed out at the end of 6 days immersion in 3X-SBF (prior to the 30 days immersion in 1X-SBF), and after the long-term immersion at the end of 30 days in 1X-SBF. During this time, the formation of CaP, HA and salt-like precipitates on the sample surfaces as well as the released ion amounts from the metals were expected to affect the weight of the samples. The results presenting the changes of sample weights are given in Table 4.

**Table 4:** Weight Changes of Samples after Long-Term Immersion Test in 1X-SBF

	Weight (g) of sample after immersed in 3X-SBF for 6 days		Weight (g) of sample after immersed in 1X-SBF for 30 days		Difference in grams		
Patterns	1st group	2nd group	1st group	2nd group	1st group	2nd group	Average increase amounts
**1**	0.43250	0.42900	0.43300	0.42950	**0.00050**	**0.00050**	**0.00050g**
**2**	0.44750	0.44200	0.44867	0.44450	**0.00117**	**0.00250**	**0.00183g**
**3**	0.43250	0.44450	0.43300	0.44650	**0.00050**	**0.00200**	**0.00125g**
**4**	0.43450	0.44850	0.43533	0.44900	**0.00083**	**0.00050**	**0.00067g**
**5**	0.44450	0.43700	0.44500	0.43750	**0.00050**	**0.00050**	**0.00050g**

When metallic implants are in contact with bodily fluids, wear behavior characteristics and weights can change during their interaction with the fluids.^11^ It is among the obtained results that bioactive precipitate deposition on the modified sample surfaces were promoted over immersion time. Similar studies conducted with stainless steel supported the results of the current study such that, the sample weight increases over time since the rougher surfaces provided a more favorable environment for precipitate deposition,^16,19^ and therefore, the sample weights increase with the formation of the oxide layer.^57^Table 4 results are also in agreement with the findings given in Figure 8 such that higher ion release occurred from the control sample which was accompanied by less deposition as compared to the patterned samples therefore less increase in weight was observed. The higher ion release rates from samples with patterns 4 and 5 where less particle deposition was observed as compared to patterns 2 and 3 also support the results of Table 4. The sample with pattern 2 exhibited the highest increase in weight among all of the samples. This increase can be explained and supported by the results of the studies of Kose (2018)^19^ and Sukuroglu et al. (2021)^60^ where higher amounts of particle depositions are evidenced in Figure 5 and Figure 6 and reduction of ion release properties can again be correlated with the more intense oxide layer formation and bioactive apatite precipitation. Therefore, as in agreement with the previous results, the findings of the weight change analysis also confirm that the samples modified with the microdeformation pattern 2 exhibited the most promising bioactivity and protectiveness against ion release among the tested samples.

**Figure 9:**
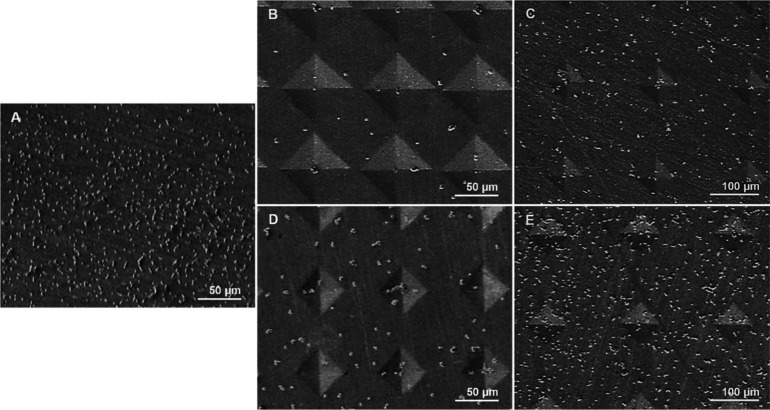
FE-SEM images of cultured and incubated *C. albicans* for 24 h on sample surfaces. A: Control/Pattern 1, B: Pattern 2, C: Pattern 3, D: Pattern 4, E: Pattern 5.

After discussion of the bioactivities of the microdeformation applied surfaces, the biofilm formation behaviors of the *C. albicans* yeast species were examined. Images of FE-SEM examinations of the samples incubated for 24 h following yeast cultivation are given in Figure 9.

**Figure 10:**
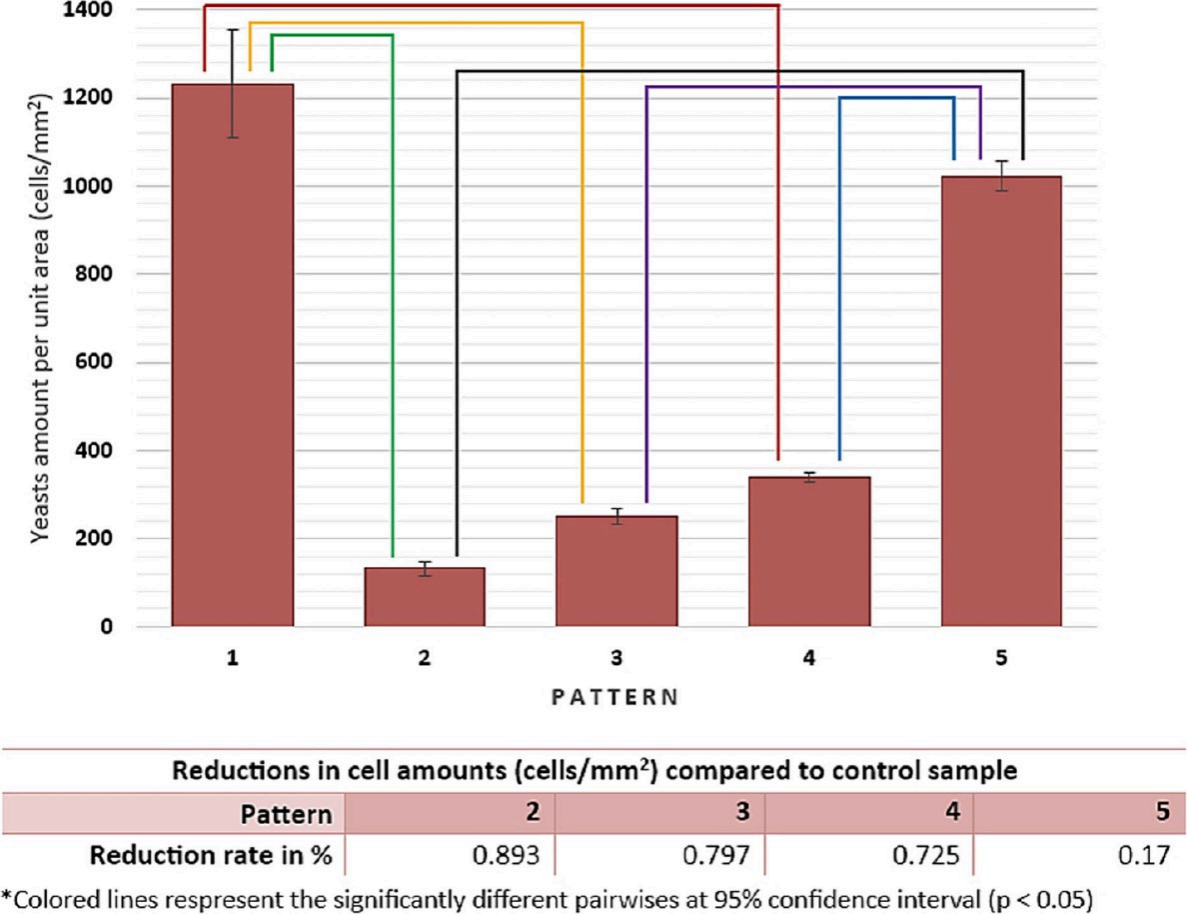
Calculated yeast cell amounts per unit area by python and their reduction rates compared to control sample after 24 h of incubation. (Results were statistically evaluated via ANOVA and compared with Tukey test at 95% confidence interval (*p* < 0.05)).

The images in Figure 9 indicated that microdeformation applied to material surfaces reduced the *C. albicans* proliferation and adhesion behaviors as compared to the control sample. In order to discuss the biofilm formation abilities of *C. albicans* on the microdeformation pattern and its relation to the surface parameters in more detail, cell densities per unit area were calculated using the FE-SEM images. First of all, yeast adhesion and proliferation behaviors on the control sample were measured with the help of the Image-J program from its SEM images. Once the binary images were obtained after various appropriate optimizations, the ratio of the cell covered area to the total surface area were calculated for all samples by the coded python program, which is based on the counting of white-black pixels. The ratios were calculated as the percent of the surface covered by cells to the total surface area. The obtained *C. albicans* densities per unit area results and the comparison of each microdeformation applied surface with the control sample as the percent (%) reduction in cell densities are given in Figure 10.

According to the results given in Figure 9 and Figure 10, the lowest cellular adhesion and proliferation were obtained on pattern 2, and comparatively, the highest cellular activities were obtained as expected in the control group. Following the control group, pattern 5 exhibits the second highest cell density among the microdeformation applied samples. The yeasts that have hypea, pseudohypea, and extracellular polymeric matrix like-morphologic structures are stated as mature biofilm indicators in the literature.^42,44^ Although 24 h of incubation is a sufficient amount of time for the formation of these morphologic structures in biofilm formation, in this study, these kinds of yeast structures were not detected on any of the samples. The aforementioned evidence showed that the 316L stainless steel is an advantageous material due to its low-rate of biofilm formation; moreover, microdeformation patterns induced great resistance to biofilm formation. It is known that biofilm formation is affected by various surface properties of the material. Surface topography, distance between repeating surface units, and depth of the indents preliminarily affect surface roughness, dislocation density, hydrophilicity, and surface energy. Those factors also closely affect biofilm formation and antibiofilm properties.^46^ According to the obtained results, deeper and closely spaced indents that result in higher surface roughness, surface energy, hydrophilicity, and dislocation density, which is obtained with pattern 2, comes forward as the most promising pattern for inhibiting the biofilm formation. Moreover, former studies report that surface energy increase beyond a certain level may impede cell proliferation, which can be another contributing factor to the decreased number of *C. albicans* on the sample with pattern 2.^51^

*C. albicans* type yeast cells, which were preferred in the current study to examine the biofilm formation mechanism and properties, preferably adhere to hydrophobic surfaces (contact angle > 90°), thus enhane biofilm formation.^39,75,76^ For the current samples, it was observed that surface hydrophobicities decreased with the application of microdeformation patterning (Figure 3). Consequently, biofilm formation decreased for each microdeformed sample as compared to the control sample due to the increased hydrophilic properties of the manipulated surfaces. *C. albicans* species have hydrophobic proteins, membrane, and compositions in its cellular structure that are normally responsible for binding the cell on the surfaces to ease cellular attachment.^42,44,45,77^ Therefore, these structures are not suitable to attach on hydrophilic surfaces, as also evident in this study. This information is supported by the findings given in Figure 4 and Figure 9, where increased hydrophilicity is shown to inhibit to cellular attachment and hinder cell proliferation. When the patterned samples are compared among themselves, it was observed that the lowest cell proliferation and attachment was obtained on pattern 2, which exhibited one of the most hydrophilic surfaces. The biofilm formation results are in agreement with the literature such that hydrophilic surface properties of pattern 2 inhibit the biofilm formation tendency. Pattern 3 exhibits relatively higher contact angles as compared to patterns 4 and 5 due to the previously explained Cassie-Bexter effect. The biofilm formation behavior when patterns 2 and 4, as well as patterns 3 and 5 are compared within themselves, and the distance factor becomes the more effective parameter influencing cellular attachment and proliferation. Moreover, when the patterns that have almost the same average roughness (e.g., patterns 2 and 3) were compared with each other, cell densities were observed to be quite distinct. It can be suggested that surface parameters such as dislocation density, roughness, and surface energy are not the only effective parameters for biofilm formation, but the distance between indents and indent depth are also quite important. Specifically, for samples with the same indent depth, lower cellular adhesion and proliferation were observed when indents are closer, which was previously explained by roughness, and the parameters which are related to roughness, such as dislocation density, surface energy, and hydrophilicity. In addition to this effect, the current findings also evidence that deeper indents promoted the improvement of surface related properties such as roughness and its correlated mechanisms.

Eventually, considering all of the experimental studies mentioned above, pattern 2 and its microdeformation parameters (close indent spacing and deeper indents caused an increase in roughness and thus higher dislocation density close to the surface, higher surface energy, and hydrophilicity) promoted the bioactivity via CaP deposition and protective layer formation. Moreover, these patterning parameters reduced the biofilm formation tendency via inducing a hydrophilic surface. In the future, patterning of metallic implant surfaces with the consideration of the specific material’s chemical and physical properties, as well as application of well-characterized methods similar to the microdeformation parameters used in the current study, can be used to enhance the bioactivity, antibiofilm, and biocompatibility properties of implants.

## 4 Conclusion

This study was carried out with 316L stainless steel, which is a widely preferred biomedical alloy for orthopedic implants due to its advantageous properties. Microdeformation patterns were applied on the sample surfaces by forming indents of different patterns, in a controlled manner with a Vicker’s microhardness testing device. Surface properties such as average surface roughness, dislocation density close to the surface, surface energy, and wettability changed as a result of the manipulation of microdeformation patterning parameters such as indent depth and spacing and thus topographical characteristics. The effects of the manipulated surface properties on the CaP deposition, protectiveness against ion release, and antibiofilm formation behaviors were evaluated. Surface roughness, dislocation activities close to the surface, surface energy, and hydrophilicity increased for all the microdeformation applied samples as compared to the control sample. In order to understand the effects of modified surface property variations on bioactivity, static immersion tests in a densified SBF environment were applied. The pattern exhibiting the highest surface energy, dislocation density, and hyrophilicity, specifically pattern 2, led to the highest amount of apatite-like structure deposition and more intense oxide layer formation, which yielded the most positive results in terms of promoting bioactivity and osseointegration and therefore contributed the most to implant biocompatibility among the formed patterns. Since the deposition of CaP-rich particles and the oxide layer reduced the amount of ions released from the metal, they also contributed to the biocompatibility of the material. Also, toxic, allergenic, carcinogenic, or harmful levels of ion release were not observed from any of the tested samples. *C. albicans* biofilm formation ability was also inhibited by the application of microdeformation patterns on the sample surfaces. Relatively lower numbers of cells were detected on all the microdeformation applied samples as compared to the control sample. Thus, it was deduced that the infection and invasiveness risk, which may be caused by biofilm formation, could also be lowered by the microdeformation surface modification. In line with the aforementioned results, the surface properties obtained by the application of the microdeformation parameters of pattern 2, specifically larger and deeper indents with close spacing, yielded the most promising results. Overall, it can be concluded that the application microdeformation to obtain controlled and regular patterns with optimum patterning parameters as in this study provides an innovative solution to osseointegration issues encountered in metallic implant materials by increasing surface energy, dislocation density, roughness, and hydrophilicity in a controlled manner. Therefore, the obtained results provide useful information for the design and development of surface modification methods to promote osseointegration, inhibit biofilm formation, and thus, improve biocompatibility for various metallic biomaterials.
